# Carotid and cerebrovascular disease in symptomatic patients with type 2 diabetes: assessment of prevalence and plaque morphology by dual-source computed tomography angiography

**DOI:** 10.1186/1475-2840-9-91

**Published:** 2010-12-18

**Authors:** Ci He, Zhi-gang Yang, Zhi-gang Chu, Zhi-hui Dong, Heng Shao, Wen Deng, Jing Chen, Li-qing Peng, Si-shi Tang, Jia-he Xiao

**Affiliations:** 1 Department of Radiology, West China Hospital, Sichuan University, Chengdu, Sichuan 610041, PR China; 2 Department of Radiology, PLA Chengdu Military Area Command General Hospital, Chengdu, Sichuan 610083, PR China; 3 State Key Laboratory of Biotherapy, West China Hospital, Sichuan University, Chengdu, Sichuan 610041, PR China

## Abstract

**Background:**

Plaque morphology directly correlates with risk of embolism and the recently developed dual-source computed tomography angiography (DSCTA) may help to detect plaques more precisely. The aim of our study was to evaluate the prevalence and morphology of carotid and cerebrovascular atherosclerotic plaques in patients with symptomatic type 2 diabetes mellitus (DM) by DSCTA.

**Methods:**

From July 2009 to August 2010, DSCTA was prospectively performed in 125 consecutive patients with symptomatic type 2 DM. We retrospectively analyzed plaque type, distribution, and extensive and obstructive natures were determined for each segment for all patients.

**Results:**

Atherosclerotic plaques were detected in 114 (91.2%) patients. Relatively more noncalcified (45%) and calcified (39%) plaques and less mixed (16%) plaques were observed (p < 0.001). Noncalcified plaques were found mainly in the intracranial arteries (81.8%), mixed plaques in the intracranial arteries (25.2%) and intracranial internal carotid artery (ICA) (56.1%). Calcified plaques were found mainly in the intracranial ICA (65.9%) and extracranial arteries (28.2%) (for all, p < 0.001). Extension of plaques from the 1^st ^to 5^th ^segments was observed in 67 (58.8%) patients and from the 6^th ^to 10^th ^segments in 35 (30.7%) patients. The most common site of all detected plaques was the cavernous segment. Regarding stenosis, there were significantly more nonobstructive than obstructive stenosis (91% vs. 9%, p < 0.001).

**Conclusion:**

DSCTA detected a high prevalence of plaques in patients with symptomatic type 2 DM. A relatively high proportion of plaques were noncalcified, as well as with nonobstructive stenosis. The distribution of plaques was extensive, with the cavernous portion of ICA being the most common site.

## Background

The number of people with diabetes mellitus (DM) in 2010 is estimated to be 285 million, which was approximately 7% of the adult world population [1]. Macrovascular disease, characterized by atherosclerotic changes in large blood vessels, is the major cause of morbidity and mortality (80%) in type 2 DM [2]; its major clinical effects are seen in the coronary arteries (angina, myocardial infarction), lower extremities (gangrene), and carotid arteries (stroke). The overall relative risk of stroke is 1.5-3 times greater in patients with DM [3]. Recurrent stroke is also twice as frequent in patients with DM [4]. More importantly, short- and long-term mortalities after stroke are significantly greater in patients with DM [5]. This underscores the need to develop practical approaches for detecting cerebrovascular disease (CeVD) at an early stage before clinical complications occur.

Although digital subtraction angiography (DSA) is the gold standard for assessing degree of stenosis [6], it cannot precisely predict plaque composition. Furthermore, DSA is an expensive technique and requires direct arterial catheterization, which makes it unacceptable for some patients [7]. Therefore, other noninvasive modalities such as MR imaging (MRI), computed tomography (CT), and duplex ultrasound are more frequently applied, wherein each offers some potential for plaque analysis. To our knowledge, the characteristics of carotid and cerebrovascular plaques detected by the recently developed dual-source CT angiography (DSCTA) in patients with symptomatic type 2 DM have not been discussed systematically. The purpose of this study was to evaluate the prevalence and morphology of carotid and cerebrovascular atherosclerotic plaques by DSCTA in a large cohort of patients with symptomatic type 2 DM.

## Methods

### Study patients

Within a 14-month period from July 2009 to August 2010, we retrospectively analyzed 125 consecutive patients with symptomatic type 2 DM who were clinically suspected with stenosis of the carotid and intracranial arteries. Inclusion criteria were patients with type 2 DM having neurologic symptoms. Symptoms were classified as follows: (1) transient symptoms, i.e., transient ischemic attack or amaurosis fugax and (2) prior stroke, i.e., any ischemic event with neurologic symptoms. Exclusion criteria were as follows: history of allergy to iodine-containing contrast medium, renal insufficiency (creatinine, ≥120 μmol/L), pregnancy, and lack of laboratory or clinical data. All patients underwent DSCTA of the carotid and cerebrovascular arteries. The following baseline demographics, laboratory findings, and medical history were obtained: age, gender, serum cholesterol level, serum HbA1c level, history of DM, hypertension, daily smoking, and cerebral infarction (CI). DM was diagnosed based on a random plasma glucose level ≥11.1 mmol/L or a fasting plasma glucose level ≥7.0 mmol/L. Hypertension was defined as treatment with antihypertensive agents at the time of admission, systolic blood pressure ≥140 mm Hg, or diastolic blood pressure ≥90 mm Hg. Subjects were classified as smoking if they smoked at least a cigarette per day in the year before the study. CI, including lacunar infarct and territorial infarct, was confirmed by CT or MRI.

### CT protocols

All examinations were performed with a DSCTA scanner (SOMATOM Definition, Siemens Medical Solutions, Forchheim, Germany) using a standardized enhanced carotid and cerebrovascular artery imaging protocol. The scanning parameters in the dual-source mode were as follows: 330-ms gantry rotation time, tube A (140 kV, 55 mAs) and tube B (80 kV, 230 mAs) with a collimation of 2 × 64 × 0.6 mm and pitch 0.65. The delay before CT acquisition after injection was determined using bolus tracking software, and a circular region of interest (ROI) for attenuation measurement was placed in the right common carotid artery (CCA). As soon as the signal intensity in ROI reached a threshold of 100 HU, data acquisition was started. A nonionic contrast medium (80-100 mL; iopamidol, 370 mg iodine/mL; Bracco Sine Pharmaceutical Corp. Ltd., Shanghai, China) was administered immediately followed by 40 mL of saline chaser solution was administered through an 18-gauge intravenous antecubital catheter at a flow rate of 6 mL/s with a dual-head power injector (Stellant; Medrad, Indianola, PA, USA). CT data was acquired in a caudocranial direction from the aortic arch to the mid-skull.

Image reconstruction was performed at another 3 D image analysis workstation (Syngo-Imaging, Siemens, Medical Solution System, Forchheim, Germany) with the following parameters: a soft convolution kernel, slice thickness of 0.75 mm, and increment of 0.4 mm. The DSCTA reader was permitted to utilize any or all available postprocessing image reconstruction algorithms, including maximal intensity projection, multiplanar reformat, curved planar reformat, or volume rendered technique and dual energy direct bone removal CTA (DE-BR-CTA).

### Imaging analysis

All images were evaluated by 2 experienced observers unaware of the clinical history of the patients. In case of disagreement, a joint reading was performed and a consensus decision was reached.

**Figure 1:**
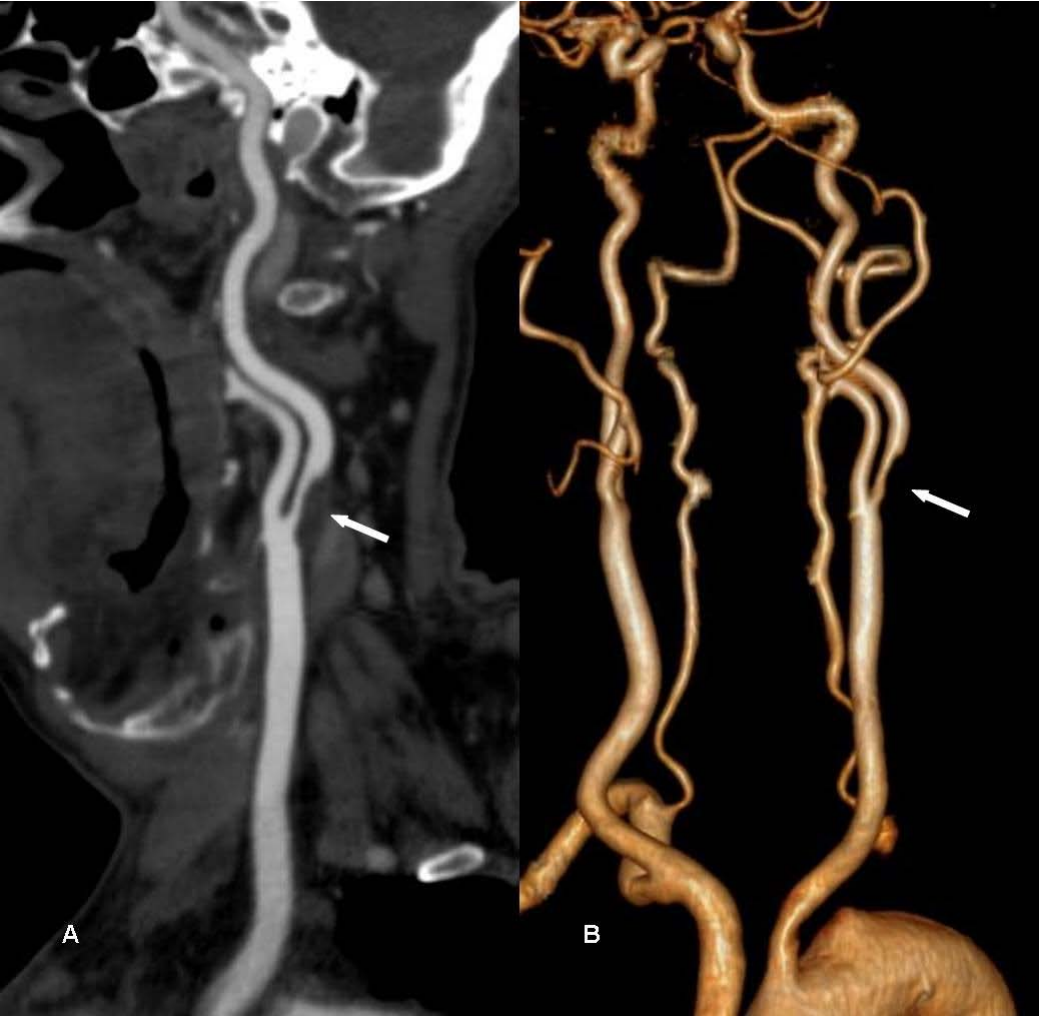
**CTA images of a 51-year-old man with 13 years diabetes**. A, a stenosis ≥70% in left common carotid artery proximal segment is caused by a non-calcified plaque (solid arrow) as evidenced using curved planar reformat (CPR). B, volume-rendered reconstruction (VRT) displays overview of arteries and stenosis (solid arrow).

**Figure 2:**
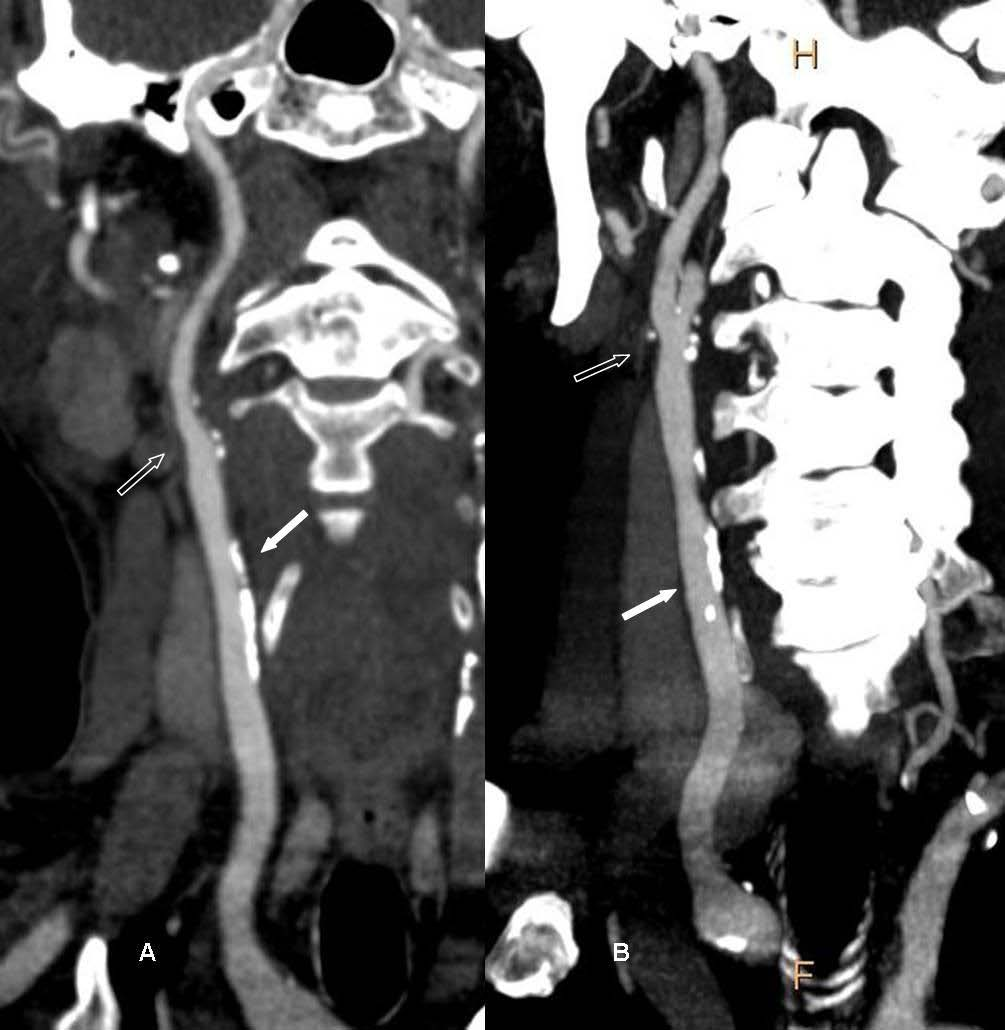
**Diffuse plaques and stenoses of a 62-year-old woman with 15 years diabetes**. A, CPR shows diffuse hard plaques in right common carotid artery (solid arrow) and mixed plaques in right internal carotid artery proximal segment (open arrow), and both resulted 25% stenoses. B, maximum intensity projection (MIP) reflects overview of the diffuse plaques and stenoses.

In order to improve reproducibility of the results, the analysis was performed on a segmental basis. According to the criteria of North American Symptomatic Carotid Endarterectomy Trial (NASCET) [8], carotid and cerebrovascular vessels were divided into 40 segments including CCA, carotid bifurcation (CB), external carotid arteries, internal carotid arteries (ICA) (C1-C7), extracranial vertebral artery (eVA), intracranial vertebral artery (iVA), basilar artery (BA), anterior cerebral artery (A1, A2), middle cerebral artery (M1, M2), posterior cerebral artery (P1, P2), anterior communicating artery (ACoA), and posterior communicating artery (PCoA). These segments were also divided into 3 categories, including extracranial arteries (CCA, CB, CA, C1, and eVA), intracranial ICA (C2-C7), and intracranial arteries (iVA, BA, A1, A2, M1, M2, P1, P2, ACoA, and PCoA). Only segments with a diameter >1.5 mm (as measured on DSCTA) were included. Three types of plaques (Figure 1 and 2) were determined using the following classification proposed by Ballotta et al. [9]: (1) noncalcified plaques, i.e., plaques with a density <50 HU; (2) calcified plaques, i.e., plaques with a mean attenuation of 130 HU or greater; and (3) mixed plaques, i.e., plaques with a mean attenuation of 50-129 HU. The grade of stenosis was rated according to the NASCET criteria [8], and all carotid and cerebrovascular arterial stenosis was scored from 0-4 according to the following scale: 0%, 1%-29%, 30%-69%, 70%-99%, and 100%. Plaques were classified as obstructive or nonobstructive based on a 70% threshold of luminal narrowing. Each vessel was analyzed on at least 2 imaging planes, 1 parallel and 1 perpendicular to the course of the vessel. Vessel diameters were measured perpendicular to the vessel course. For each patient the number of diseased segments, plaque type, and degree of stenosis were determined and recorded.

### Statistical analysis

Continuous variables were expressed as the mean ± standard deviation. Categorical variables were presented as percentages. Comparisons between different types of plaque, degree of stenosis, and plaque location were performed by the nonparametric chi-square test. All statistical analyses were performed using SPSS version 16.0 (SPSS Inc., Chicago, IL). A p value < 0.05 was considered statistically significant.

## Results

### Patient characteristics

Carotid and cerebrovascular DSCTA was performed in 125 patients with symptomatic type 2 DM. A total of 4723 arterial segments were included in this analysis, while 277 vascular segments were not evaluated because of deficiency or diameter <1.5 mm. Baseline characteristics of the 125 patients who were analyzed are summarized in Table 1.

**Table 1:** Characteristics of our study population

Characteristics	data
Age (years)	66.36 ± 12.38 (38-92)
Duration of diabetes (years)	6.4 ± 5.8 (0.3-32)
Men	78 (62.4)
Smoking	43 (34.4)
Duration of smoking (years)	28.5 ± 13.1 (0.2-60)
Hypertension	85 (68)
Duration of hypertension (years)	11.1 ± 10.7 (0.3-50)
CI	81 (64.8)
Plaques	114 (91.2)
Triglyceride (mmol/L)	1.6 ± 0.8 (0.4-6.4)
Cholesterol (mmol/L)	4.3 ± 1.2 (2.1-10.9)
HDL-C (mmol/L)	1.2 ± 0.4 (0.3-2.5)
LDL-C (mmol/L)	2.5 ± 1.1 (0.2-7.8)
HbA1c (%)	8.1 ± 1.8 (5.9-11.9)
Insulin	20 (16.0)
Oral agent	89 (71.2)
Nephropathy	22 (17.6)
Retinopathy	72 (57.6)

Results are given as mean ±standard deviation (range) or n (percentage). Normal range (Triglyceride: 0.29-1.83 mmol/L; Cholesterol: 2.8-5.7 mmol/L; HDL-C: >0.9 mmol/L; LDL-C:<4.0 mmol/L; HbA1c: 4.5%-6.1%). CI = cerebrovascular infarction; HDL-C = high density lipoprotein cholesterol; LDL-C: low density lipoprotein cholesterol.

### Plaque prevalence, composition, and distribution

DSCTA revealed 11 (8.8%) patients without CeVD and 114 (91.2%) patients with CeVD. A total of 658 segments with plaque were identified, of which 296 (45%) were noncalcified, 107 (16%) were mixed, and 255 (39%) were calcified (p < 0.001). Regarding plaque distribution, noncalcified plaques were mainly located in the intracranial arteries (81.8%), mixed plaques in the intracranial arteries (25.2%) and intracranial ICA (56.1%), and calcified plaques in the intracranial ICA (65.9%) and extracranial arteries (28.2%) (for all, p < 0.001; Table 2). The distribution of plaques was extensive; 67 (58.8%) had 1^st^-5^th ^diseased segments, and 35 (30.7%) 6^th^-10^th ^diseased segments. The most common site of all detected plaques in the patients was the cavernous portion of ICA (131/658, 19.9%) followed by C5 (70/658, 10.6%) and CB (68/658, 10.3%).

**Table 2:** Comparison of stenosis and location between different plaques

Characteristics	Noncalcified	Mixed	Calcified	*P*
**Type**	296(45)	107(16)	255(39)	< 0.001
**Stenosis**				
Mild	233(78.7)	69(64.5)	141(55.3)	< 0.001
Moderate	52(17.6)	29(27.1)	75(29.4)	< 0.001
Severe	10(3.4)	7(6.5)	33(12.9)	< 0.001
Occlusion	1(0.3)	2(1.9)	6(2.4)	0.097
**Location**				
Extracranial artery	39(13.2)	20(18.7)	72(28.2)	< 0.001
Intracranial ICA	15(5.0)	60(56.1)	168(65.9)	< 0.001
Intracranial artery	242(81.8)	27(25.2)	15(5.9)	< 0.001

Results are given as n (percentage).

ICA = internal carotid artery.

### Arterial stenosis

Of the segments with plaques, mild, moderate, severe stenosis, and occlusion were observed in 443 (67%), 155 (24%), 51 (8%), and 9 (1%) segments, respectively (p < 0.001). In general, 598 (91%) showed nonobstructive CeVD and 60 (9%) showed obstructive CeVD (p < 0.001). There were different grades of stenosis in different plaques. Noncalcified plaques led to 233 (78.7%) mild, 52 (17.6%) moderate, 10 (3.4%) severe, and 1 (0.3%) occlusion. Mixed plaques led to 69 (64.5%) mild, 29 (27.1%) moderate, 7 (6.5%) severe, and 2 (1.9%) occlusions. Calcified plaques resulted in 141 (55.3%) mild, 75 (29.4%) moderate, 33 (12.9%) severe, and 6 (2.4%) occlusions (Table 2). There was a trend that noncalcified plaque resulted in a higher incidence of nonobstructive lumen narrowing while calcified plaque resulted in a higher incidence of obstructive lumen narrowing. In total, for noncalcified, mixed, and calcified plaques, 96.3%, 91.6%, and 84.7% were nonobstructive (p < 0.001), respectively.

## Discussion

Type 2 DM is a disease with both metabolic and vascular components [10]. Patients with DM should always control their risk factors and recognize the signs and symptoms of potentially fatal complications as early as possible. Plaque morphology directly correlates with the risk of embolism and occlusion [11], thus detecting plaques at an early stage is of great clinical importance. As a noninvasive modality, multidetector CT angiography (MDCTA) offers some potential for plaque analysis [12,13]. DSCTA offers many advantages over conventional MDCTA. First, DSCTA can differentiate materials by analyzing their attenuation differences [14]. As a result, bone and calcified plaque can be removed from vessels with iodine contrast. Second, reduction in radiation dose is an important characteristic of DSCTA. Zhang et al. [15] reported that compared with conventional bone-subtraction CTA, DE-BR-CTA showed a 60% reduction in radiation dose for dual-source CTA, avoiding the additional preliminary unenhanced CT acquisition. Third, DSCTA has faster scan acquisition and higher spatial resolution, allowing DSCT acquisition with minimal patient motion registration artifact and better visualization of mixed and noncalcified plaques in small vessels [14,16]. Furthermore, DSCTA has advanced postprocessing methods, especially easy-to-use bone-removal algorithms for direct visualization of complex vasculature, and high quality DSA-like imaging [16].

In our study, the metabolic disorders in patients with symptomatic DM predominantly included hypertension, glucose metabolism abnormalities, and dyslipidemia. A high incidence and wide distribution of plaques were identified in the present study. The heavy plaque burden in patients with DM was probably because there were more cerebrovascular risk factors resulting from metabolic disorders that have been described as the cause of atherosclerosis of the carotid and cerebrovascular arteries [17,18]. Guidelines for treatment of specific conditions with respect to metabolic disorders are being developed [19] and should be promoted by healthcare educators and providers.

Regarding the composition of atherosclerotic plaques in patients with type 2 DM, we found that there were relatively more noncalcified and calcified plaques and less mixed plaques, which was consistent with previous studies [1,20]. These observations may suggest a more rapid development of atherosclerosis in the presence of DM, with faster progression from noncalcified lesions to completely calcified lesions [20]. A faster progression of atherosclerosis has also been suggested previously on the basis of event rates in patients with DM undergoing nuclear perfusion imaging [21]. Interestingly, a recent study using MDCT demonstrated that a higher proportion of mixed plaque was found in diabetics than in nondiabetics [22]. However, the number of study subjects was small and they were all asymptomatic for cardiac symptoms. There is no clear explanation for this difference. Despite many controversies, noncalcified plaques have been suggested as potentially vulnerable to trigger plaque rupture or embolism [23]. It is important to evaluate the CeVD potential and treat the remediable plaques in a timely manner.

The present study demonstrated that patients with symptomatic type 2 DM showed a significantly higher prevalence of nonobstructive lesions (91%), confirming the findings of previous studies [1,24,25]. Using 64-slice MDCT, Scholte et al. [1] showed that coronary plaques of patients with type 2 DM were primarily nonobstructive (82%). Saely et al. reported a similar relationship between DM and nonobstructive plaques by invasive coronary angiography [24]. It has been suggested that plaque rupture may occur frequently in nonobstructive plaques [24]. This finding is of clinical importance since these plaques may be vulnerable to rupture and may be related to the high morbidity and mortality in patients with DM.

As was shown in this study, the noncalcified plaques were primarily located in the intracranial arteries, mixed plaques in the intracranial arteries and intracranial ICA and calcified plaques in the intracranial ICA and extracranial arteries. A similar distribution of calcified plaques has been shown in another study using 16-slice MDCT [26]. In our study, a higher incidence of noncalcified plaque detected in intracranial vessels could be attributed to better visualization of noncalcified plaques in small vessels by DSCTA. In addition, we found a relatively high incidence of mixed plaque in the vessels located next to the cranium, due to the high spatial resolution of DSCTA with easy-to-use bone-removal algorithms that provides a direct visualization of complex vasculature [16].

In our study, the most common site of plaques was the cavernous portion of ICA. Wojak et al. [27] confirmed this, reporting that intracranial atherosclerotic stenosis typically occurred in the petrous cavernous siphon segments of ICA. Furthermore, Masuoka et al. [28] investigated the cause of development of atheromatous plaque around the cavernous portion of ICA using serial 3-mm sections of 32 intracranial ICA segments obtained from 50 cadavers, and found that the external elastic lamina disappeared in the cavernous portion of ICA; intimal thickening of ICA frequently appeared in the horizontal segment of the cavernous portion of ICA, which was the most common site of stenosis. Change in the elasticity of the arterial wall in the cavernous portion was suggested to be an important factor in the process of atherosclerosis in the intracranial ICA.

Outlining the significance of our study, the prevalence and morphology of carotid and cerebrovascular atherosclerotic plaques in patients with symptomatic type 2 DM by DSCTA has been systematically reported for the first time. The DSCT findings, which depended on a large study sample with cutting-edge technology, accurately reflects characteristics of plaque and stenosis in patients with symptomatic type 2 DM, and can be used to conduct a further treatment plan.

There were several limitations to this study. First, this study is a single centre, retrospective study. A study with a larger patient population from various centers is warranted to confirm these data. Second, the visualization of noncalcified plaques by DSCT is limited by plaque size; smaller plaques located predominantly in the smaller arteries may therefore be difficult to identify accurately with the current generation of CT scanners.

## Conclusion

DSCTA detected a high prevalence of CeVD in patients with symptomatic type 2 DM. A relatively high proportion of plaques was noncalcified and calcified, primarily leading to nonobstructive stenoses. The distributions of plaques were different and extensive, with the most common site being the cavernous segment of ICA.

## Competing interests

The authors declare that they have no competing interests.

## Authors' contributions

All authors participated in the design and coordination of the study, reviewed the analysis and took part in writing the manuscript. They also read and approved the final manuscript.
